# Artificial Radionuclides Database in the Pacific Ocean: HAM Database

**DOI:** 10.1100/tsw.2004.15

**Published:** 2004-03-15

**Authors:** Michio Aoyama, Katsumi Hirose

**Affiliations:** Geochemical Research Department, Meteorological Research Institute, Nagamine 1-1, Tsukuba, Ibaraki 305-0052, Japan

**Keywords:** database, artificial radionuclides, marine environment, plutonium

## Abstract

The database “Historical Artificial Radionuclides in the Pacific Ocean and its Marginal Seas”, or HAM database, has been created. The database includes Sr, Cs, and Pu concentration data from the seawater of the Pacific Ocean and its marginal seas with some measurements from the sea surface to the bottom. The data in the HAM database were collected from about 90 literature citations, which include published papers; annual reports by the Hydrographic Department, Maritime Safety Agency, Japan; and unpublished data provided by individuals. The data of concentrations of Sr, Cs, and Pu have been accumulating since 1957—1998. The present HAM database includes 7737 records for Cs concentration data, 3972 records for Sr concentration data, and 2666 records for Pu concentration data. The spatial variation of sampling stations in the HAM database is heterogeneous, namely, more than 80% of the data for each radionuclide is from the Pacific Ocean and the Sea of Japan, while a relatively small portion of data is from the South Pacific. This HAM database will allow us to use these radionuclides as significant chemical tracers for oceanographic study as well as the assessment of environmental affects of anthropogenic radionuclides for these 5 decades. Furthermore, these radionuclides can be used to verify the oceanic general circulation models in the time scale of several decades.

### INTRODUCTION

Chemical tracers are useful tools to understand the physical and biogeochemical processes in the ocean. Tritium, CFCs, and others have been used as transient tracers of physical processes, such as mixing and advection, in the ocean. The distributions of chemical tracers have been used for validation of global oceanic circulation models.

Artificial radionuclides ($^{90}$Sr, $^{137}$Cs, and $^{239,240}$Pu) that have been injected into the ocean by global fallout, released from the nuclear processing plants and others, might be also significant transient tracers. Although the concentrations of these radionuclides in seawater have been determined for the assessment of environmental affects of radionuclides for these 5 decades, they have not been used for oceanographic purposes. One of the exceptions are the data obtained from GEochemical Ocean SECtions Study (GEOSECS) in the 1970s[1].

We think a large number of the concentrations of these radionuclides in seawater, which were published in variety of research fields and governmental reports, might be useful for oceanographic studies as well as the study of artificial radionuclides in the marine environment. We, then, constructed the database “Historical Artificial Radionuclides in the Pacific Ocean and its Marginal Seas”, or HAM database. The database includes $^{90}$Sr, $^{137}$Cs, and $^{239,240}$Pu concentration data from the seawater in the Pacific Ocean and its marginal seas with some measurements from the sea surface to the bottom. The data of concentrations of $^{90}$Sr, $^{137}$Cs and $^{239,240}$Pu have been accumulating since 1957–1998. The Marine Environment Laboratory of the IAEA has also created and maintained a database on marine radioactivity, the Global Marine Radioactivity Database (GLOMARD)[2]. GLOMARD consists of radionuclide concentration data for seawater, sediment, biota, and suspended matter. During the first phase of GLOMARD, the work concentrated on the Sea of Japan and the Barents and Kara Seas in the arctic in connection with the investigation of radioactive waste dumping sites and their impact on the marine environment. Some of the data in the GLOMARD and our HAM database may overlap, especially those from the Sea of Japan and western North Pacific. The HAM database, however, focuses on the radionuclides concentration in seawater in the Pacific Ocean and its marginal seas, so further data exchange between the two databases will make the both databases more powerful. In this paper, we present the HAM database in detail and discuss its features.

### OBJECTIVES

One of the major objectives of the HAM database is to reconstruct the history of artificial radionuclides in the oceans. Since our interest is mainly in the Pacific Ocean and its marginal seas, our effort to collect data sources was concentrated in these sea areas. We expect that the artificial radionuclides are good transient tracers of oceanographic studies in the Pacific Ocean and its marginal seas. $^{137}$Cs, which is a soluble radionuclide, is a good tracer for water mass movement. $^{239,240}$Pu, which is a typical particle reactive radionuclide, could also be used for biogeochemical studies of trace metals, including radionuclides, in the ocean. The HAM database can be applied to validate the results of the 3D simulations using ocean global circulation models.

Some results of the studies using the HAM database are already published. A typical example is a study on the formation of deep waters in the Sea of Japan. It provided new information that the formation of deep waters occurred in the 1990s based on the analysis of the $^{137}$Cs concentration data in the database (Fig. 2 in [3]). The temporal variation of the $^{137}$Cs concentration in the surface water in the North Pacific during these 4 decades was examined by Hirose and Aoyama (Figs. 1, 2, 3, 6, and 12 in [4]). Aoyama and Hirose also examined the temporal variation of the $^{137}$Cs inventory in the North Pacific, then, increases of the $^{137}$Cs inventory at the lower latitude of 10–20° N in the North Pacific were found in the 1970s and 1980s. It is concluded that surface and subsurface southward transports were considered as the source of this increasing $^{137}$Cs inventory (Figs. 3 and 4 in [5]). In the studies of temporal variation of $^{137}$Cs and $^{239,240}$Pu in the Pacific Ocean using an oceanic general circulation model, the HAM database was also used to verify the results of the model calculation by Tsumune et al.[6,7].

### DATABASE CONSTRUCTION

#### Sources of the Data in this Database

We collected about 90 literature citations to construct the HAM database during these 7 years. As shown in Table 1, the sources of data are as follows:

1. Geochemical Research Department, Meteorological Research Institute, JAPAN (hereafter GRD/MRI) data from the scientific papers in the Pacific Ocean since 1960[8,9,10,11,12,13,14,15,16,17,18,19,20,21,22].
2. Health and Safety Laboratory, now Environmental Measurement Laboratory, USA (hereafter HASL/EML**)** reports[23,24,25].
3. Scientific papers, reports, and books 1960–1995[26,27,28,29,30,31,32,33,34,35,36,37,38,39,40,41,42,43,44,45,46,47,48,49,50,51,52,53,54,55,56,57,58,59,60].
4. The annual reports of the Hydrographic Department/Maritime Safety Agency/Japan during the period from 1963–2000[61,62,63,64,65,66,67,68,69,70,71,72,73,74,75,76,77,78,79,80,81,82,83,84,85,86,87,88,89,90,91,92,93].
5. Unpublished data obtained from the authors[94,95,96,97,98,99,100].

The numbers of data of each radionuclide from each source are also shown in Table 1.

This database includes 7737 records for $^{137}$Cs concentration data, 3972 records for $^{90}$Sr concentration data, and 2666 records for $^{239,240}$Pu concentration data. For the $^{137}$Cs concentration data, the major contributors are Folsom's work in the 1960s and the 1970s and the long-term work by the Hydrographic Department, Maritime Safety Agency, Japan during 1963–2000. About 3000 of the total 7737 data of the $^{137}$Cs concentration are available from HASL/EML reports[23,24,25]. The Hydrographic Department, Maritime Safety Agency, Japan contributed about 1000 data of the $^{137}$Cs concentration during 1963–1998. For the $^{90}$Sr concentration data, the major contributors are Livingston's work in the GEOSECS project and the long-term work by the Hydrographic Department, Maritime Safety Agency during 1963–1998. About 700 of the total 3972 data of the $^{90}$Sr concentration are available from Livingston et al.[39]. The Hydrographic Department, Maritime Safety Agency contributed about 1000 data of $^{90}$Sr concentration during 1963–1998. For the $^{239,240}$Pu data, the major contributors are Livingston et al.[39], Wong et al.[60], and Noshkin et al.[56]. The Hydrographic Department, Maritime Safety Agency also contributed about 500 data of the $^{239,240}$Pu concentration during 1989–2000.

### Handling of Original Data in the HAM Database

All of the data in the publications/reports are treated as follows:

a. Input original data in original unit with data originator name.
b. The values in the original unit are converted to the values in the SI unit, Bq $m^{-3}$.
c. Create Microsoft Access file. Microsoft Access is a desktop database software that operates in a user desktop environment such as Microsoft Windows 98, Windows Me, or Windows XP.
d. Create PostgreSQL file. PostgreSQL is an object-relational database management system (ORDBMS) based on POSTGRES, Version 4.2, developed at the University of California at Berkeley Computer Science Department. PostgreSQL is an open-source descendant of this original Berkeley code. It supports SQL92 and SQL99 and offers many modern features.
e. Web database access for data search.

Throughout the handling of the data, the data originator name, the name of the reference literature, and original values with original unit were kept. When counting errors were reported in the original literature, those were also kept throughout the handling. To identify the original literature, a unique code, of which field name in the database is MRIREFNO, is given for each literature and added to each record. For each profile, a unique number, of which field name is profileID, is also given. A code to identify that the data are in a certain profile, of which field name is PS, is given as P. A code S at the field PS is given for the concentration data of the surface water only without deeper layer sampling. Keeping these original and created data throughout the process allows us to maintain the database more easily. Users of the database can also see the original values and unit together with the values in SI by the database creator.

**TABLE 1**

**Data Sources and Number of the Data Archived in the Database for Each Radionuclide**

| MRIREFNO | $^{137}$Cs | $^{90}$Sr | Pu | Ref. | MRIREFNO | $^{137}$Cs | $^{90}$Sr | Pu | Ref. |
|---|---|---|---|---|---|---|---|---|---|
| Aoyama1995 | 67 | 0 | 39 | [8] | msa1992 | 64 | 68 | 24 | [85] |
| Aoyama2001 | 34 | 0 | 0 | [9] | msa1993 | 64 | 64 | 13 | [86] |
| Bourlat1992 | 3 | 0 | 0 | [26] | msa1994 | 64 | 63 | 13 | [87] |
| Bourlat1995 | 0 | 0 | 22 | [27] | msa1995 | 150 | 152 | 102 | [88] |
| Bourlat1996 | 54 | 54 | 51 | [28] | msa1996 | 113 | 114 | 69 | [89] |
| Broecker1966 | 49 | 43 | 0 | [29] | msa1997 | 96 | 98 | 55 | [90] |
| Broecker1968 | 123 | 78 | 0 | [30] | msa1998 | 89 | 90 | 63 | [91] |
| Folsom1960a | 12 | 0 | 0 | [31] | msa1999 | 86 | 87 | 63 | [92] |
| Folsom1960b | 4 | 0 | 0 | [32] | msa2000 | 101 | 101 | 63 | [93] |
| Folsom1968 | 1141 | 0 | 0 | [23] | Ikeuchi1999 | 30 | 30 | 30 | [37] |
| Folsom1970 | 712 | 0 | 0 | [24] | Imai1973 | 0 | 0 | 9 | [38] |
| Folsom1975 | 62 | 0 | 0 | [33] | Livingston1985 | 79 | 709 | 552 | [39] |
| Folsom1979 | 1188 | 0 | 0 | [25] | Livingston1987 | 70 | 0 | 12 | [40] |
| Fowler1983a | 0 | 0 | 5 | [34] | Livingston2000 | 15 | 0 | 14 | [96] |
| Fowler1983b | 0 | 0 | 8 | [94] | Miyake1960 | 0 | 9 | 0 | [14] |
| Fowler1991 | 0 | 0 | 20 | [35] | Miyake1961 | 6 | 6 | 0 | [15] |
| Hirose1992 | 3 | 0 | 4 | [10] | Miyake1962 | 9 | 9 | 0 | [16] |
| Hirose1999 | 23 | 23 | 23 | [11] | Miyake1963 | 3 | 3 | 0 | [17] |
| Hirose2000 | 11 | 0 | 110 | [95] | Miyake1968 | 0 | 0 | 15 | [18] |
| Hirose2001 | 12 | 0 | 52 | [12] | Miyake1978 | 0 | 0 | 37 | [19] |
| Hirose2002 | 11 | 0 | 56 | [13] | Miyake1988 | 126 | 0 | 46 | [20] |
| Hong1999 | 21 | 0 | 22 | [36] | Miyao1998 | 8 | 0 | 31 | [21] |
| msa1963 | 11 | 11 | 0 | [61] | Nagaya1964 | 12 | 12 | 0 | [41] |
| msa1965 | 51 | 48 | 0 | [62] | Nagaya1965 | 32 | 31 | 0 | [43] |
| msa1966 | 40 | 40 | 0 | [63] | Nagaya1970 | 53 | 59 | 0 | [44] |
| msa1967 | 51 | 55 | 0 | [64] | Nagaya1976 | 34 | 99 | 0 | [45] |
| msa1968 | 49 | 53 | 0 | [65] | Nagaya1981 | 77 | 77 | 0 | [46] |
| msa1969 | 49 | 50 | 0 | [66] | Nagaya1984 | 94 | 97 | 73 | [47] |
| msa1970 | 56 | 59 | 0 | [67] | Nagaya1987 | 104 | 70 | 78 | [48] |
| msa1971 | 47 | 51 | 0 | [68] | Nagaya1993 | 46 | 0 | 24 | [49] |
| msa1976 | 114 | 115 | 0 | [69] | Nakanishi1990 | 0 | 0 | 117 | [50] |
| msa1977 | 49 | 49 | 0 | [70] | Nakanishi1995 | 0 | 0 | 55 | [51] |
| msa1978 | 38 | 38 | 0 | [71] | Noshkin1974 | 4 | 0 | 2 | [97] |
| msa1979 | 37 | 37 | 0 | [72] | Noshkin1976 | 5 | 0 | 5 | [52] |
| msa1980 | 51 | 53 | 0 | [73] | Noshkin1978 | 21 | 0 | 24 | [53] |
| msa1981 | 60 | 61 | 0 | [74] | Noshkin1979 | 0 | 0 | 23 | [54] |
| msa1982 | 70 | 75 | 0 | [75] | Noshkin1981 | 0 | 0 | 5 | [55] |
| msa1983 | 43 | 43 | 0 | [76] | Noshkin1987 | 9 | 0 | 0 | [98] |
| msa1984 | 72 | 76 | 0 | [77] | Noshkin1999 | 196 | 39 | 266 | [56] |
| msa1985 | 57 | 62 | 0 | [78] | Noshkin2000 | 13 | 0 | 8 | [99] |
| msa1986 | 73 | 78 | 0 | [79] | Pillay1964 | 1 | 1 | 2 | [57] |
| msa1987 | 67 | 65 | 0 | [80] | Delfanti2000 | 99 | 34 | 88 | [58] |
| msa1988 | 80 | 78 | 0 | [81] | Saruhashi1975 | 231 | 176 | 0 | [22] |
| msa1989 | 72 | 72 | 24 | [82] | Shirasawa1968 | 83 | 85 | 0 | [59] |
| msa1990 | 66 | 66 | 24 | [83] | Wong1971 | 1 | 0 | 3 | [100] |
| msa1991 | 56 | 56 | 12 | [84] | Wong1992 | 131 | 0 | 210 | [60] |
| | | | | | **Total** | **7737** | **3972** | **2666** | |

## FEATURES OF THE HAM DATABASE

### Geographical Distribution

The total numbers of the data in the HAM database for the three major radionuclides in each sea area are summarized in Table 2. In the database, sea area code is given in each data. The geographical distributions for each decade from the 1950s to the 1990s are shown in Figs. 1 to 5, respectively. Since our effort was concentrated in the Pacific Ocean and its marginal seas, more than 80% of the data for each radionuclide are for the Pacific Ocean and the Sea of Japan in the HAM database as shown in Table 2. In the 1950s, a few $^{137}$Cs and $^{90}$Sr concentration data at the western North Pacific near Japan were available as shown in Fig. 1. The $^{239,240}$Pu concentration data did not exist in this decade in HAM database. The number of $^{137}$Cs concentration data increased abruptly in the North Pacific in the 1960s, especially in 1966–1968 as shown in Fig. 6. The station locations of $^{137}$Cs concentration data covered well over the Pacific Ocean, while $^{90}$Sr concentration data and $^{239,240}$Pu concentration data showed still less and sparsely, respectively, in the 1960s (Fig. 2). In the 1970s (Fig. 3), the number of $^{137}$Cs concentration data decreased, however, the station locations of $^{137}$Cs concentration data covered well over the Pacific Ocean. The station locations of $^{90}$Sr concentration data and $^{239,240}$Pu concentration data covered the North Pacific Ocean. The geographical distributions of $^{137}$Cs concentration data and $^{90}$Sr concentration data in the 1980s were concentrated at the western North Pacific, while that of $^{239,240}$Pu concentration data covered over the North Pacific (Fig. 4). No data area at the central and the eastern North Pacific for the three radionuclides was seen in the 1990s (Fig. 5).

**TABLE 2**
**Total Number of the Data in Oceans and Marginal Seas**

| Sea Area | Area Code | $^{137}$Cs | $^{90}$Sr | Pu |
|---|---|---|---|---|
| Eastern North Pacific | NEP | 2530 | 373 | 598 |
| Western North Pacific | NWP | 2620 | 1650 | 1009 |
| Eastern South Pacific | SEP | 510 | 115 | 63 |
| Western South Pacific | SWP | 127 | 12 | 30 |
| Sea of Japan | SOJ | 1276 | 1224 | 541 |
| Sea of Okhotsk | SOO | 21 | 16 | 35 |
| East China Sea | ECS | 48 | 41 | 3 |
| South China Sea | SCS | 16 | 0 | 1 |
| North Atlantic | NA | 238 | 287 | 171 |
| South Atlantic | SA | 135 | 190 | 109 |
| Indian Ocean | IO | 106 | 26 | 14 |
| Not assigned yet | XXX | 110 | 38 | 92 |
| **Total** | | **7737** | **3972** | **2666** |

### Temporal Variation of the Numbers for the Three Radionuclides

The temporal variation of the numbers of the data for the three radionuclides in each year is shown in Fig. 6. $^{137}$Cs and $^{90}$Sr concentration data were available since 1957, while $^{239,240}$Pu data were available since 1964. A maximum number of 1268 for the $^{137}$Cs concentration data was in 1967, while the numbers of $^{90}$Sr and $^{239,240}$Pu concentration data were 83 and 15 in the same year, respectively. This tendency to have so much $^{137}$Cs concentration data and less $^{90}$Sr and plutonium concentration data was observed during the

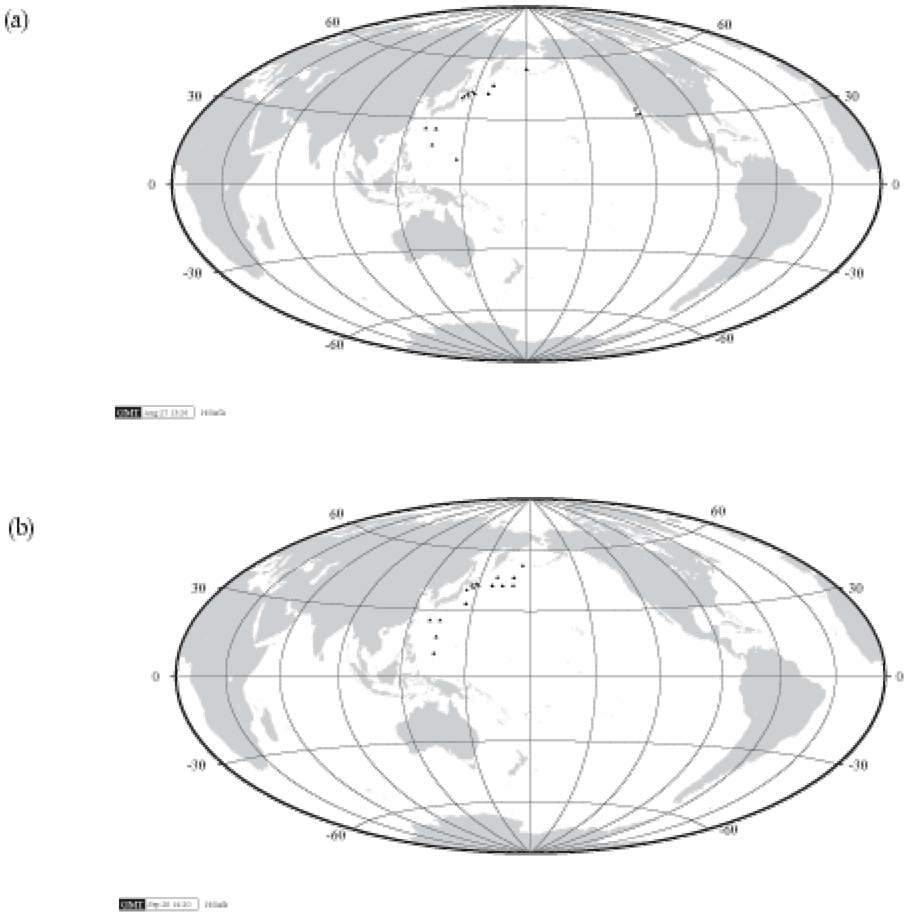

**FIGURE 1**. Geographical distribution of $^{137}$Cs and $^{90}$Sr concentration data in the 1950s in HAM database. (a)$^{137}$Cs; (b) $^{90}$Sr.

1960s. The numbers of the data for the three radionuclides became the same due to the GEOSECS project, in which the analysis for the three radionuclides was done simultaneously in the early 1970s. The numbers of the data for each radionuclide were around 100 per year throughout the 1980s and the 1990s.

### The Number of the Data for the Surface Water and the Profiles

The number of the data for surface water and profiles in each of the oceans and marginal seas are summarized in Table 3 for each radionuclide. For the $^{137}$Cs concentration data, 55.7% of the total amount of 7737 data is for surface data, then the remaining 44.3% is for the profiles. The numbers of the profile for the $^{137}$Cs is about 430. For the $^{90}$Sr concentration data, 42.7% of the total amount of 3972 is for the surface and 57.3% is for the profiles. The numbers of the profile for the $^{90}$Sr is about 320. For the $^{239,240}$Pu concentration data, 23.6% of the total amount of 2666 is for the surface and 76.4% is for the profile. The numbers of the profile for the $^{239,240}$Pu is about 240.

### Counting Error of the Data

The number of the data associated with counting error for the $^{137}$Cs concentration data is 7164, which is 92.6% of the total amount of 7737 in this database. For the $^{90}$Sr concentration data and the $^{239,240}$Pu concentration data, 98.0% of the total amount of 3972 and 96.0% of the total amount of 2666 in this database are reported with counting error.

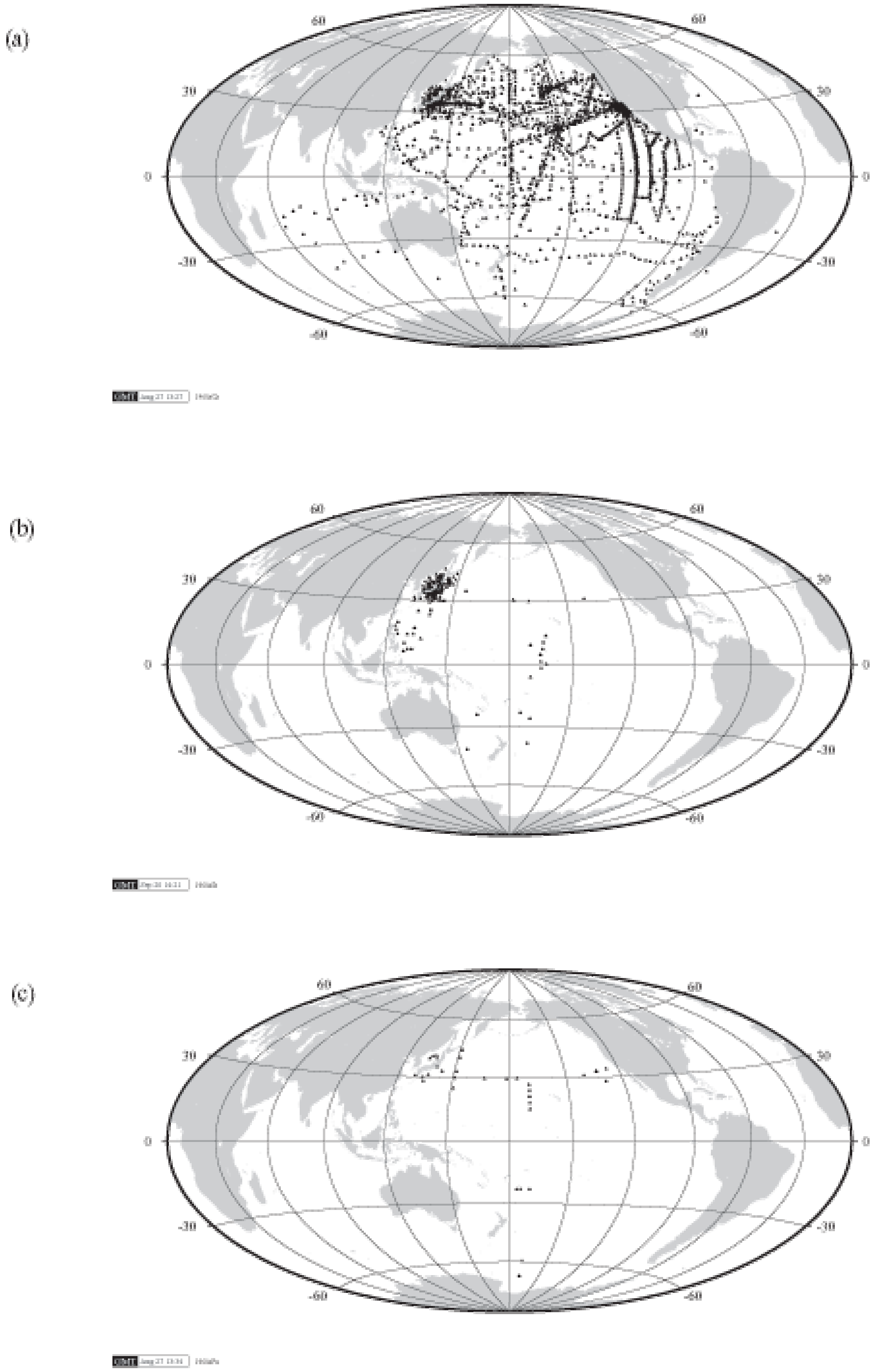

**FIGURE 2**. Geographical distribution of $^{137}$Cs, $^{90}$Sr, and $^{239,240}$Pu concentration data in the 1960s in HAM database. (a) $^{137}$Cs; (b) $^{90}$Sr; (c) $^{239,240}$Pu.

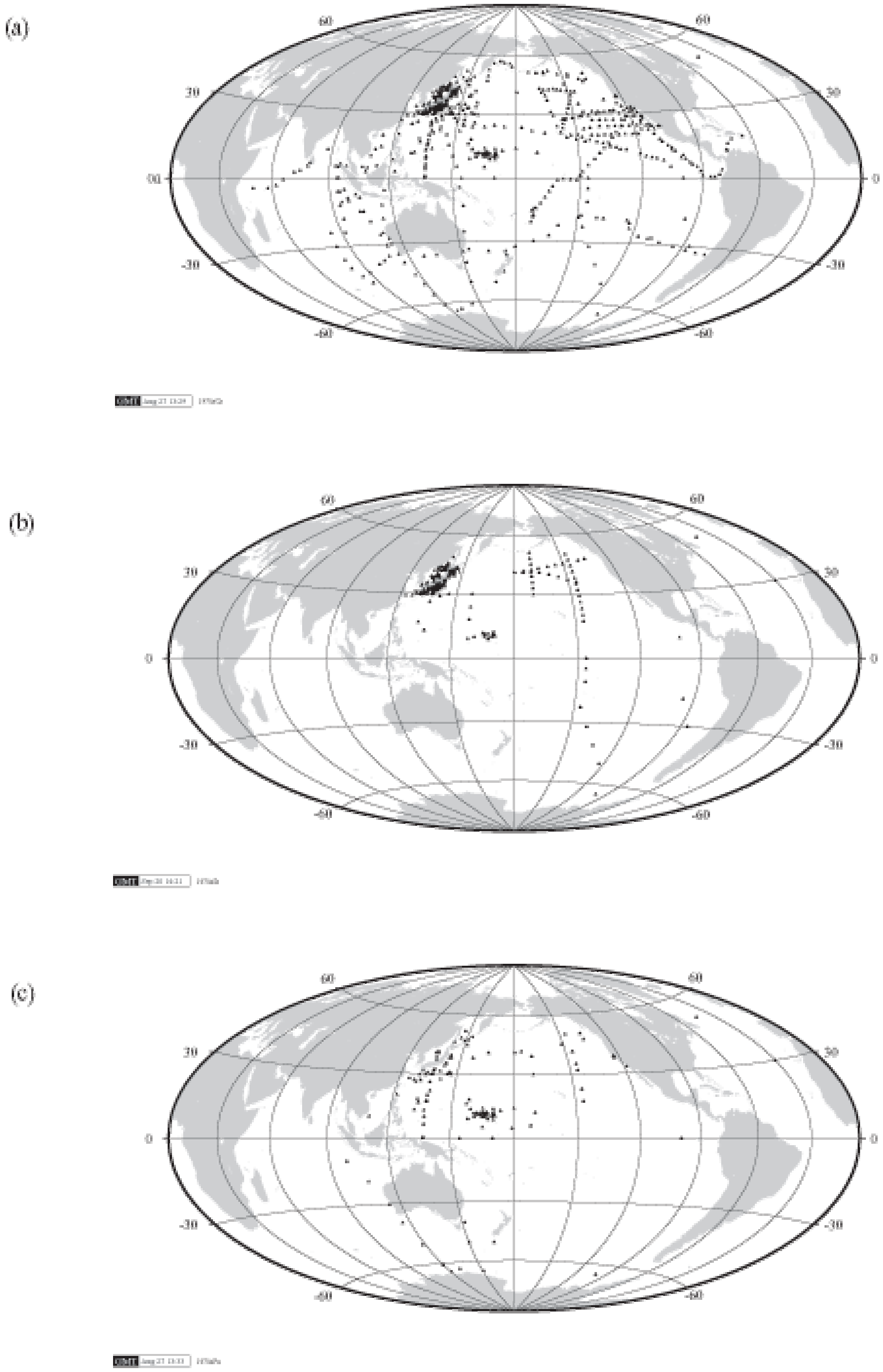

**FIGURE 3**. Geographical distribution of $^{137}$Cs, $^{90}$Sr, and $^{239,240}$Pu concentration data in the 1970s in HAM database. (a) $^{137}$Cs; (b) $^{90}$Sr; (c) $^{239,240}$Pu.

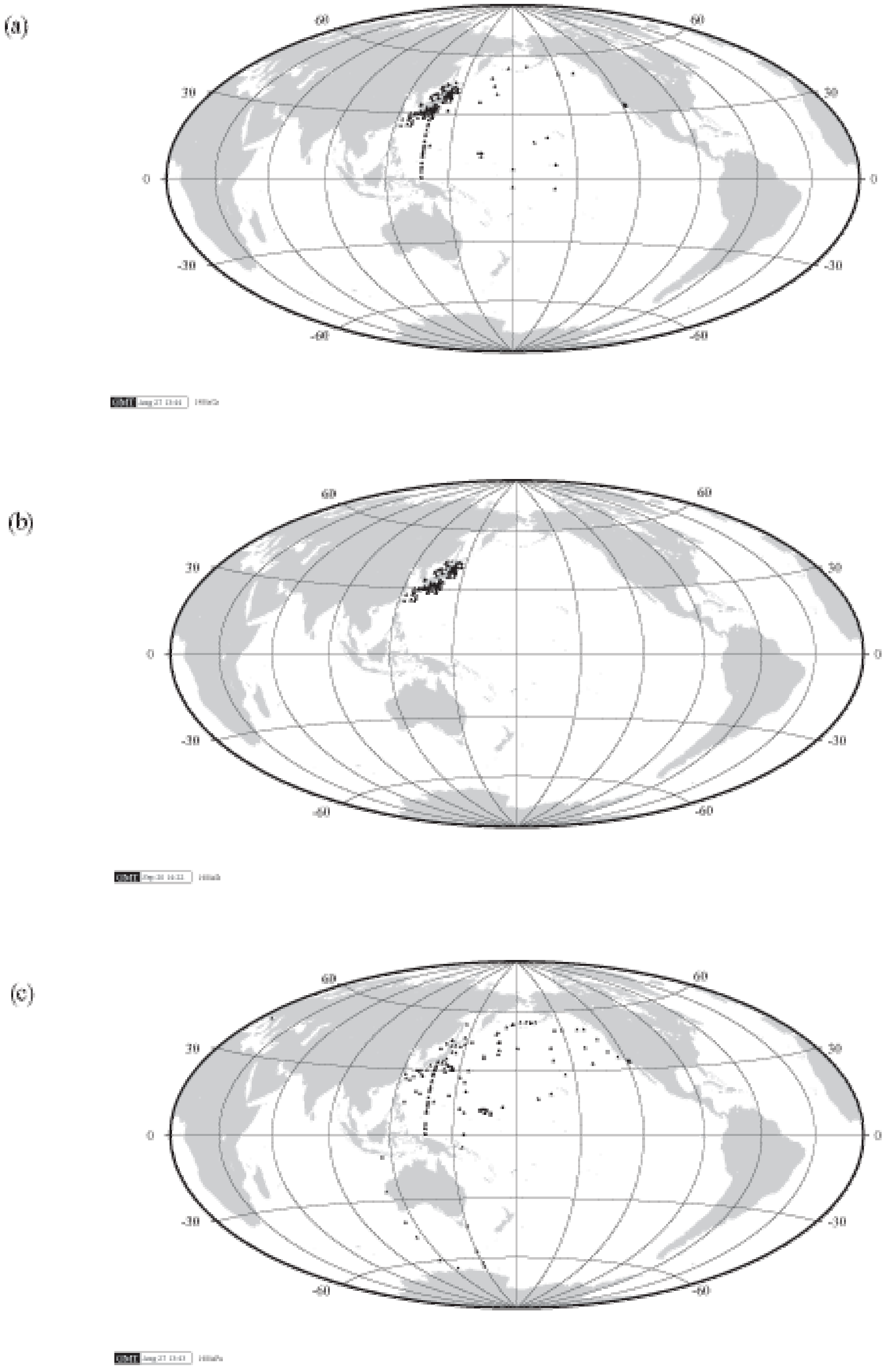

**FIGURE 4**. Geographical distribution of $^{137}Cs$, $^{90}Sr$, and $^{239,240}Pu$ concentration data in the 1980s in HAM database. (a) $^{137}Cs$; (b) $^{90}Sr$; (c) $^{239,240}Pu$.

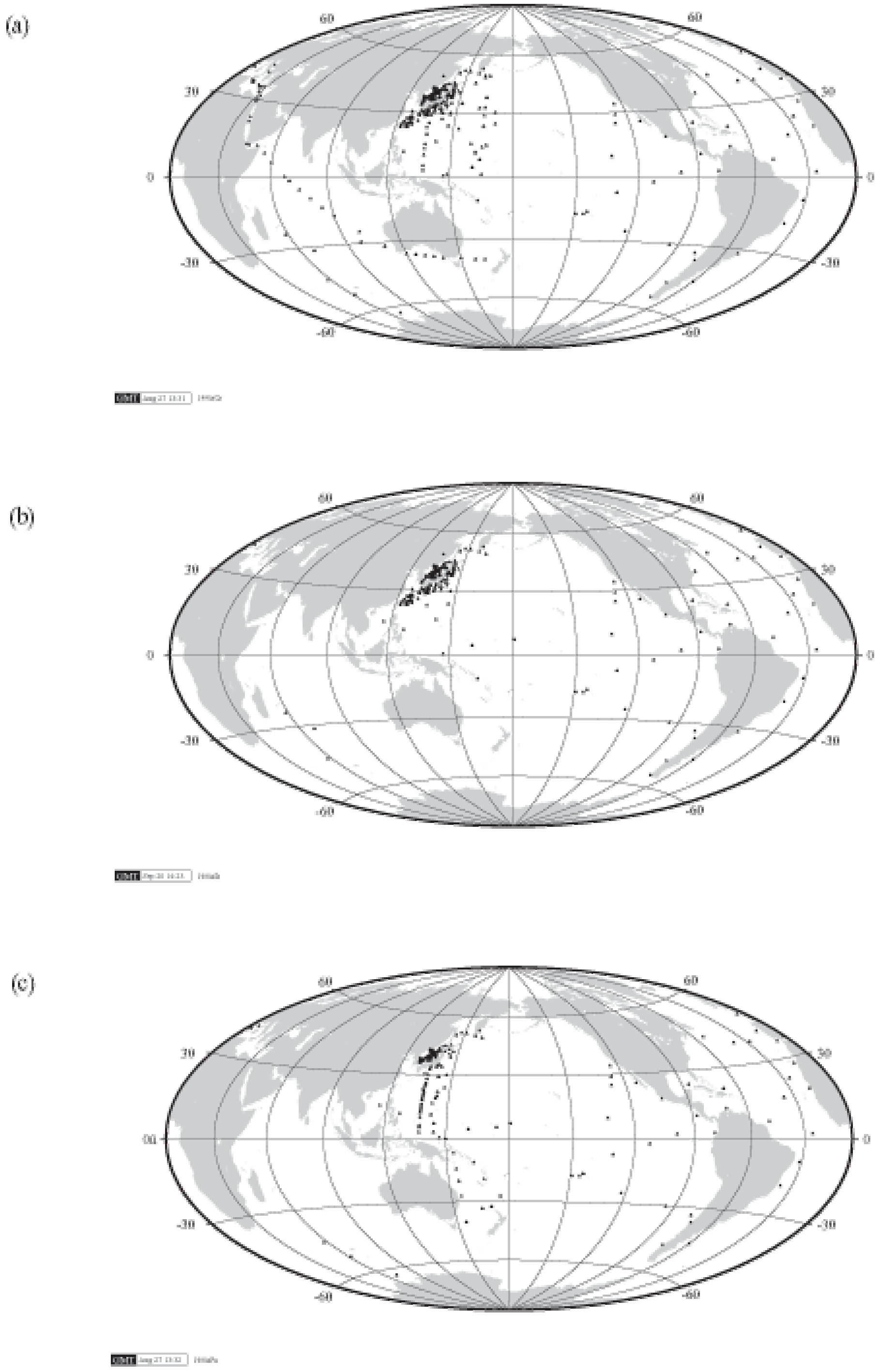

**FIGURE 5**. Geographical distribution of $^{137}Cs$, $^{90}Sr$, and $^{239,240}Pu$ concentration data in the 1990s in HAM database. (a) $^{137}Cs$; (b) $^{90}Sr$; (c) $^{239,240}Pu$.

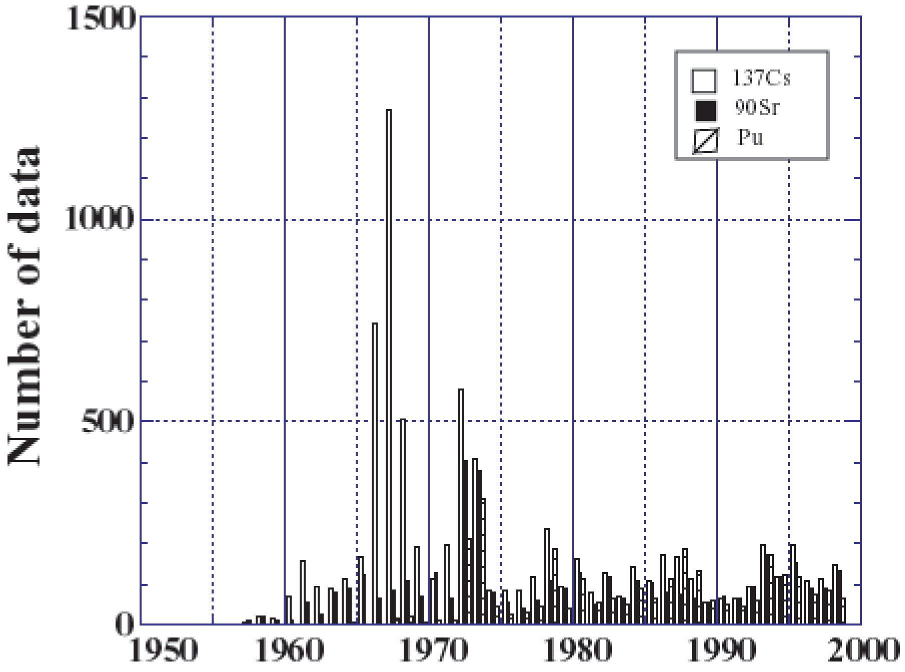

**FIGURE 6**. Number of concentration data for each radionuclides during the period from 1957–1998. Open bar: $^{137}$Cs; solid bar: $^{90}$Sr; hatched bar: $^{239,240}$Pu.

**TABLE 3**
**The Number of the Data for Surface Water and Profiles in Oceans and Marginal Seas**

| Sea Area | Area Code | $^{137}$Cs | | $^{90}$Sr | | Pu | |
|---|---|---|---|---|---|---|---|
| | | Surface | Profiles | Surface | Profiles | Surface | Profiles |
| Eastern North Pacific | NEP | 1410 | 1120 | 56 | 317 | 96 | 502 |
| Western North Pacific | NWP | 1444 | 1176 | 772 | 878 | 368 | 641 |
| Eastern South Pacific | SEP | 368 | 142 | 23 | 92 | 39 | 24 |
| Western South Pacific | SWP | 118 | 9 | 12 | 0 | 26 | 4 |
| Sea of Japan | SOJ | 738 | 538 | 715 | 509 | 56 | 485 |
| Sea of Okhotsk | SOO | 5 | 16 | 0 | 16 | 3 | 32 |
| East China Sea | ECS | 44 | 4 | 41 | 0 | 3 | 0 |
| South China Sea | SCS | 16 | 0 | 0 | 0 | 1 | 0 |
| North Atlantic | NA | 22 | 216 | 17 | 270 | 16 | 155 |
| South Atlantic | SA | 5 | 130 | 5 | 185 | 3 | 106 |
| Indian Ocean | IO | 106 | 0 | 26 | 0 | 14 | 0 |
| Not assigned yet | XXX | 36 | 74 | 30 | 8 | 4 | 88 |
| Subtotal | | 4312 | 3425 | 1697 | 2275 | 629 | 2037 |
| | | (55.7%) | (44.3%) | (42.7%) | (57.3%) | (23.6%) | (76.4%) |
| **Total** | | **7737** | | **3972** | | **2666** | |

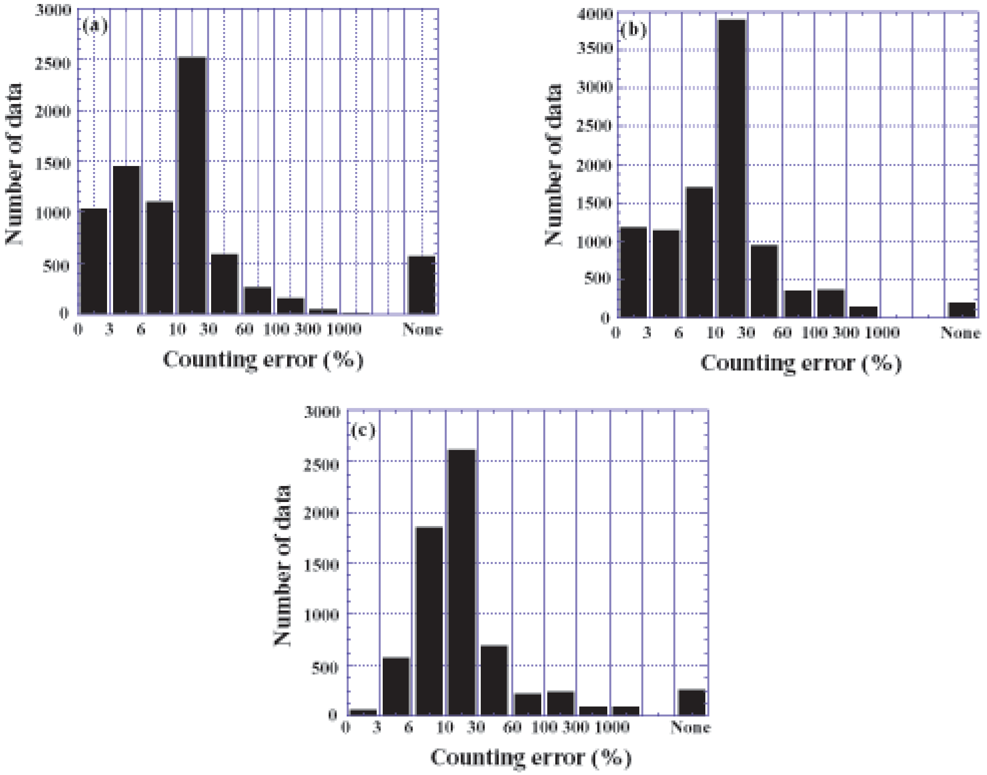

**FIGURE 7**. Number of data for each error class for each radionuclide in the database. (a) $^{137}$Cs; (b) $^{90}$Sr; (c) $^{239,240}$Pu.

For the $^{137}$Cs concentration data, the magnitude of the counting error varied within 30% in general as shown in Fig. 7(a). Of the total amount of 7164, 208 (2.9%) showed larger counting error exceeding 100%. Those larger counting errors were seen for the data in deeper layers[24,25,29,30, 36,39,47,48,75,78,79,81,83,85,91] where the $^{137}$Cs concentrations were generally very low. For the $^{90}$Sr concentration data, the magnitude of the counting error varied within 30% in general as shown in Fig. 7(b). Of the total amount of 3892, 205 (5.3%) show larger counting error, which is exceeding 100%. Those larger counting errors were seen for the data in deeper layers[29,30,39,45,47,48, 73,74,77,78,79,84,85] as well as the $^{137}$Cs concentration data. For the $^{239,240}$Pu concentration data, the magnitude of the counting error varied between 6 and 30% in general as shown in Fig. 7(c). Of the total amount of 2562, 167 (6.5%) show larger counting error, which is exceeding 100%. Those larger counting errors were seen both in shallower layers and deeper layers[47,48,54,60] .

### DATABASE DISTRIBUTION AND CITATION OF THIS DATABASE

This HAM database is freely available on request. When results or illustrations obtained using the HAM database are presented in papers, their authors should cite this paper together with a version of the HAM database used.

The description in this paper on the HAM database is based on the version U as of 29 Dec. 2001.

### CONCLUSIONS

The database regarding “Historical Artificial Radionuclides in the Pacific Ocean and its Marginal Seas”, HAM database, has been created. The data in the HAM database have been collected from about 90

literature citations, which include published papers/reports; annual reports of the Hydrographic Department, Maritime Safety Agency, Japan; and unpublished data provided by individuals. The data of the concentrations of $^{90}$Sr, $^{137}$Cs, and $^{239,240}$Pu have been accumulating since 1957–1998. The present database includes 7737 records for $^{137}$Cs concentration data, 3972 records for $^{90}$Sr concentration data, and 2666 records for $^{239,240}$Pu concentration data. The spatial variation of sampling stations in the HAM database is heterogeneous, namely, more than 80% of the data for each radionuclide is from the Pacific Ocean and the Sea of Japan, while a relatively small amount of data is from the South Pacific. This HAM database will allow us to use these radionuclides as significant chemical tracers for oceanographic studies as well as the assessment of environmental affects of anthropogenic radionuclides for these 5 decades. Furthermore, these radionuclides can be used to verify the oceanic general circulation models in the time scale of several decades.

### ACKNOWLEDGMENTS

The authors thank the scientists and researchers who published the papers used in this database. The authors also thank V.E. Noshkin, K.M. Wong, S.W. Fowler, and H.D. Livingston, who provided unpublished data for this database. The authors thank Kayono Yamamoto for her work while we were constructing the HAM database. This study is a part of the research program of "Study of long term behavior of radionuclides in the ocean" funded by MEXT (The Ministry of Education, Culture, Sports, Science and Technology).
